# Asymmetric split H-shape nanoantennas for molecular sensing

**DOI:** 10.1364/BOE.8.000395

**Published:** 2016-12-21

**Authors:** I. G. Mbomson, S. Tabor, B. Lahiri, G. Sharp, S. G. McMeekin, R. M. De La Rue, N. P. Johnson

**Affiliations:** 1 School of Engineering, University of Glasgow, Glasgow, G12 8LT, UK; 2 School of Computing and Engineering, Glasgow Caledonian University, Glasgow, G4 0BA, UK

**Keywords:** (130.6010) Sensors, (250.5403) Plasmonics

## Abstract

In this paper we report on a very sensitive biosensor based on gold asymmetric nanoantennas that are capable of enhancing the molecular resonances of C-H bonds. The nanoantennas are arranged as arrays of asymmetric-split H-shape (ASH) structures, tuned to produce plasmonic resonances with reflectance double peaks within the mid-infrared vibrational resonances of C-H bonds for the assay of deposited films of the molecule 17β-estradiol (E2), used as an analyte. Measurements and numerical simulations of the reflectance spectra have enabled an estimated enhancement factor on the order of 10^5^ to be obtained for a thin film of E2 on the ASH array. A high sensitivity value of 2335 nm/RIU was achieved, together with a figure of merit of approximately 8. Our experimental results were corroborated using numerical simulations for the C-H stretch vibrational resonances from the analyte, superimposed on the plasmonic resonances of the ASH nanoantennas.

## Introduction

**Fig. 1:**
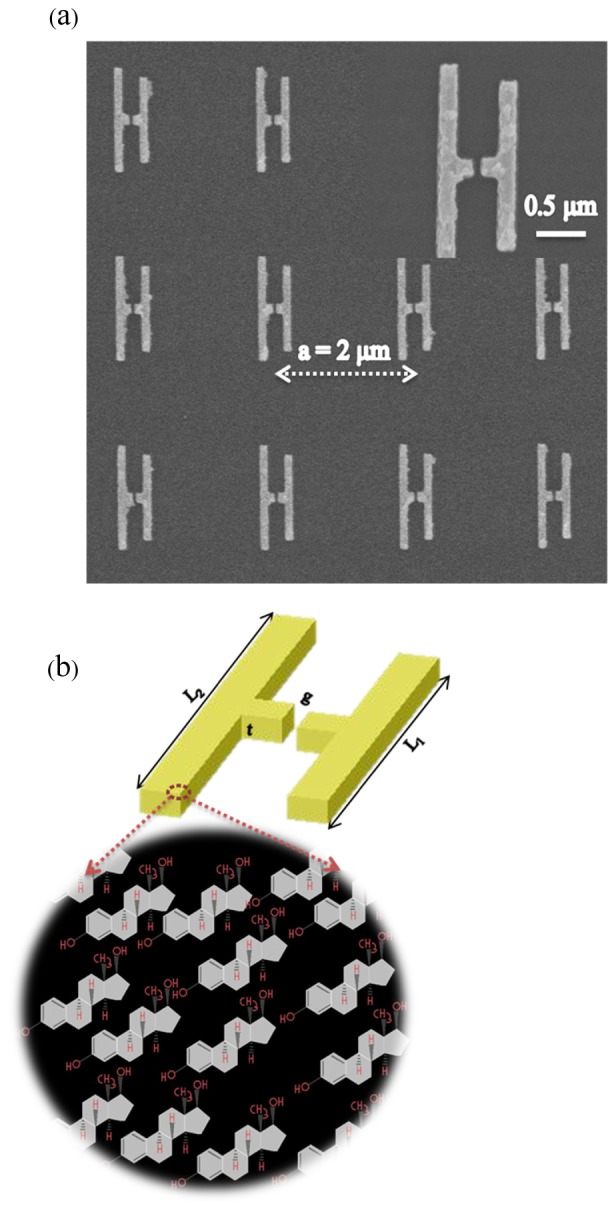
(a) Scanning electron microscope (SEM) images of ASH structure fabricated on a fused silica substrate (b) Schematic diagram of an ASH nanoantenna with arrays of two dimensional (2D) systematic molecular structure of 17β-estradiol.

Recently, there has been a rapid growth of interest in nanoantenna based sensors because of their potential application in several fields including chemistry, biology and medicine. Several studies have been reported where small amounts of analyte were detected using the plasmonic resonance produced by coupling the analyte with metallic patterns [1–5]. A suitably optimized resonant response can produce a highly sensitive and selective device based on arrays of nanoantenna [6–10]. Several methods have been employed to enhance the plasmonic oscillation of nanoantennas for easy identification of analytical compounds in different portions of the electromagnetic (EM) spectrum. In the literature, methods that have been applied include graphene-mediated surface-enhanced Raman spectroscopy (SERS) in the visible range [11], surface enhanced infrared absorption (SEIRA) spectroscopy [12–15], photothermal-induced resonance (PTIR) in the infrared [16] and surface plasmon resonance enhancement in the microwave region [17,18]. Large enhancement factors are very important for easy identification of the small amounts of analyte present in the sensor surroundings. In this paper we show enhancement of vibrational resonances obtained by depositing a thin film of 17β-estradiol (E2) as an analyte on asymmetric split H-shape (ASH) nanoantennas, as shown in Fig. 1(a)

. The asymmetric ASH structure produces double plasmonic resonance peaks. The large enhancement and sensitivity resulting from the novel ASH nanoantenna structure are attributable to the sharp edges and to the narrow slit in the middle of the nanostructure, which produce a large, but polarization-dependent, localized-enhancement of the optical electric field.

The analyte 17β-estradiol hormone, commonly referred to as E2 and shown schematically in Fig. 1(b), has a molar mass of 272.382 g/mol and is mostly responsible for controlling the development of human sex organs [19]. E2 is particularly used during preparation of in vitro fertilization and monitoring ovulation induction that are important for human reproduction. It can also be found naturally in the environment through human excreta or by anthropogenic activities [20]. The large demand for E2 for use in clinical analysis in determining the performance of the human reproductive system [19, 21,22], and its possible presence in water treatment have motivated researchers to investigate this particular analyte and report on various techniques for sensing its presence [20, 23–30]. These techniques, such as screen-printed carbon electrochemistry (SPCE) [23], gas chromatography-mass spectrometry (GC-MS) [24], gold electrode surface via under-potential deposition (UDP) [25] and aptamer-based optical fiber [26], give a modest limit of detection (LOD) and require complex experimental laboratory work. Recent development of the SEIRA method by using plasmonic resonance from nanostructures has been shown to be rapid and simple for a specific assay of 17β-estradiol [31,32]. However, the approach used excluded the evaluation of the molecular-bond vibrational resonance signature in the mid-infrared region that is exhibited by E2, with LOD values on the nanomolar scale [26, 31–39]. In this study, we show that with the new ASH structure, zeptomole LOD values can be achieved through evaluation of the C-H vibrational resonance exhibited by E2. Other researchers have evaluated the vibrational resonances of the C-H bond in organic analytes such as polydimethylsiloxane (PDMS), 1-octadecanthiol (ODT) and poly-methyl-methacrylate (PMMA) which also reveal other molecular-bond stretch resonances in the mid-infrared region [2, 40–42]. Our designed ASH nanoantennas enable a large enhancement value of 10^5^ for the C-H resonance peaks present in E2.

Among the molecular bond vibration resonances that occur in the 3 μm to 8 μm mid-infrared (MIR) region, the C-H bond has strong vibrational resonances that cover the spectrum between 3.31 μm and 3.55 μm, with double resonance peaks [41–47]. The vibrational resonance of the C-H bond present in PMMA has previously been enhanced using the SEIRA method [42] by a factor of 10^3^. Through experimental measurement and numerical simulations we have assayed E2, which we have previously shown to exhibit C = C and C-H bond stretching resonances in the mid-infrared region [45]. The C-H vibrational resonance enhancement is achieved by applying the SEIRA technique with ASH structures that have nanoscale gaps and half-wave dipole arms that are resonant at relevant wavelengths. The enhancement factor that we have obtained is greater than the values previously reported for probing and evaluation of the C-H vibrational resonances exhibited by various biochemical analytes using the SEIRA technique [42, 45,47]. Moreover, through numerical simulations we have established matching vibrational resonances to the C-H bond stretching resonance through a Lorentz-oscillator model. Through the numerical modeling for the vibrational resonances, an LOD estimate as low as a zeptomole was obtained. We show here that, for our novel ASH structure, which exhibits a nanoscale gap and multiple sharp corners, the proposed design, with a proper choice of shape and size, can produce a highly sensitive plasmonic sensor [40,46,48–52]. These features of ASH nanoantennas support high E-field enhancement while the asymmetric nature of the structure increases the sensitivity of the device by producing a larger relative shift in the plasmonic resonance peaks, in the presence of analyte [40–42].

## Experiment

Square arrays of ASH nanoantennas with asymmetric arm lengths of L_1_ = 0.9 μm and L_2_ = 1.1 μm respectively were fabricated on clean fused-silica substrates using electron beam lithography (EBL) as previously described in references [44,45]. The periodic spacing, a, between the ASH elements in the arrays was 2 μm, along both in-plane axes. The gap, g, in the ‘horizontal’ bar between the arms was chosen to be 50 nm and the patterned arm and bar widths, w, were all 100 nm, as shown in Fig. 1(a)-1(b). The thickness, t, of the gold metallization was 100 nm and a 10 nm thick titanium adhesion layer was used. The analyte 17β-Estradiol (C_18_H_24_O_2_), with a molecular mass of 272.382 g/mol, was obtained from Sigma-Aldrich. E2 was mixed with absolute ethanol and formed clear solutions, after thorough shaking, for four separate experiments with respective concentrations of 100, 10, 1 and 0.1 mg/ml, which correspond to solution molarities of 334, 36, 3.7 and 0.37 μmol/ml, respectively. A pipette was used to deposit the solution on the samples. The ethanol was allowed to evaporate, leaving an E2 thickness, for the 1 mg/ml sample, of approximately 200 nm, measured with a Dektak profilometer.

A Bruker FTIR and Hyperion microscope combination was used for the reflectance measurements, in normal incidence - firstly on arrays of as-fabricated ASH nanoantennas and then on arrays with E2 deposited over the entire array area. The double resonance peaks were produced with the electric field polarized parallel to the arms of the ASH, using a ZnSe single-crystal IR polarizer. The results from measurements on the ASH arrays were normalized with respect to the reflectivity of an Au coated mirror.

## Sensitivity

**Fig. 2:**
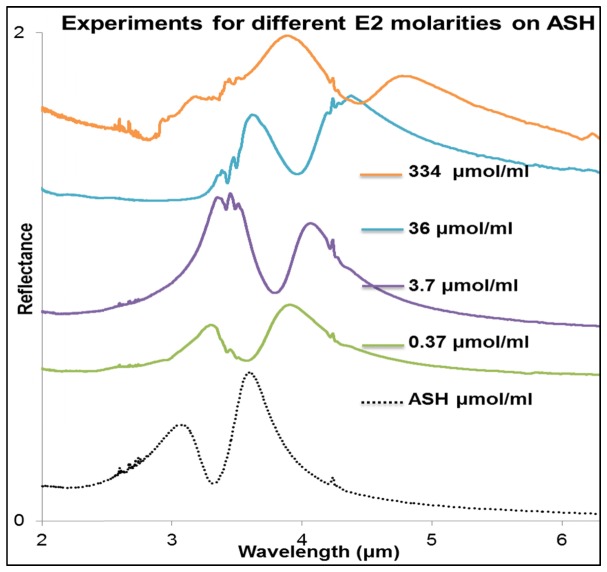
Reflectance spectra measurements with different concentrations of analyte. The plots are displaced vertically for clarity.

To demonstrate the sensing ability of the ASH nanoantenna array, we have calculated the sensitivity associated with the redshifts in the plasmonic resonance peaks produced by refractive index changes in the environment of the ASHs. The sensitivity values of the reflectance resonance for the four distinct experiments were calculated using the relationship s = Δλ/Δn nm/RIU, where Δλ and Δn are the changes in the resonance peak position and refractive index respectively [53]. The reflectance peaks shown in Fig. 2(a)

shifted from 3.586 μm, before deposition of estradiol, to 3.87, 4.06, 4.38 and 4.87 μm, respectively giving Δλ values of 286, 469, 794 and 1284 nm, respectively. For the experimental solution concentrations of 0.37, 3.7, 36, and 334 μmol/ml, sensitivity values of 520, 853, 1444 and 2335 nm/RIU, respectively were calculated for the deposited solid E2 films. These values were estimated on the basis of the change in refractive index (0.55) produced by the deposition of the 17β-estradiol film, displacing air in the region immediately above the ASH arrays.

Figure 2 shows the experimental changes in the reflection spectra for the deposited E2 films obtained from the different solution concentrations of 0.37, 3.7, 36 and 334 μmol/ml. The calculations were based on the shift in the longer wavelength resonance peak. The high sensitivity value is partly attributable to the asymmetric nature of the nanoantenna, with a dip occurring in the reflectance spectra between the resonance peaks produced by the two arms of the ASH. A full-width half-maximum (FWHM) of 310 nm was measured for the longer wavelength resonance peak at 3.586 μm. The FWHM value was used, together with the sensitivity of 2335 nm/RIU to calculate the figure of merit (FOM) via the formula FOM = sensitivity/FWHM, producing a FOM value of 7.5. These values are comparable to those in reference [40] (sensitivity 2546 nm/RIU and FOM = 8.9) however, the calculated values depend on the index of the analyte at the measured wavelength which are different. The approximate thickness of the E2 film for a concentration of 3.7 μmol/ml was measured as 200 nm.

**Fig. 3:**
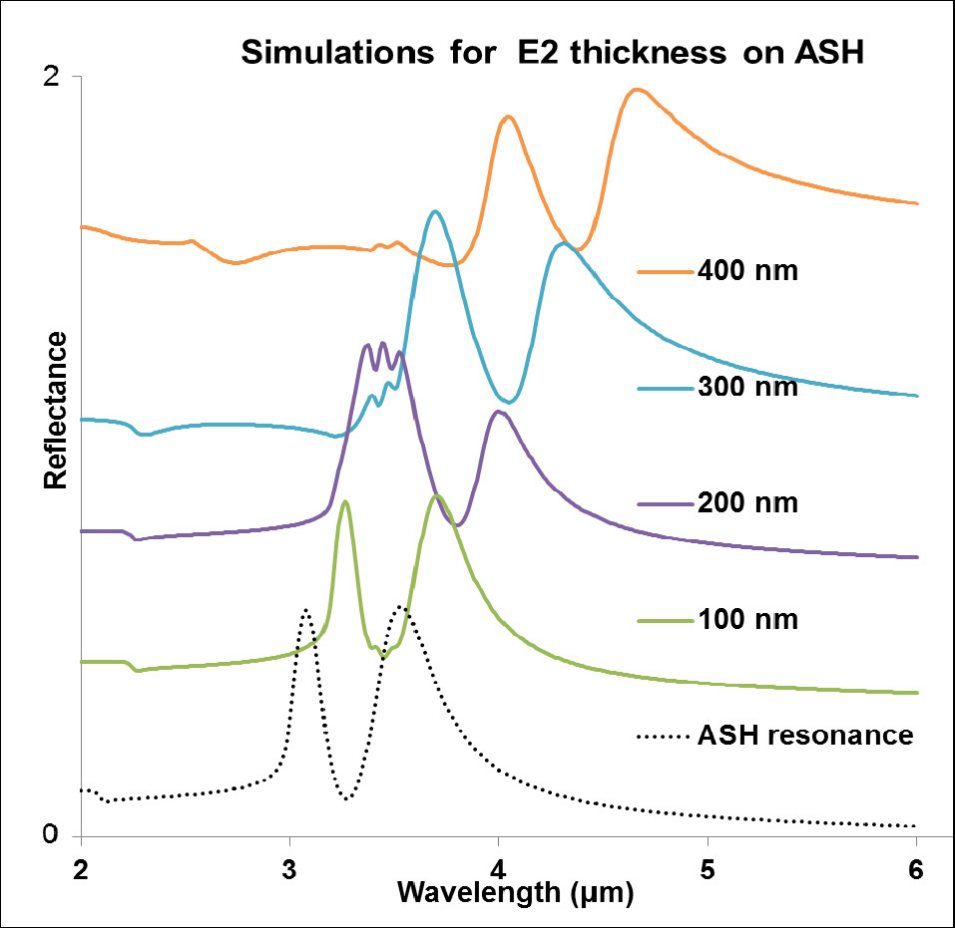
Reflectance spectra simulations with different thicknesses. The plots are displaced vertically for clarity.

Simulations covering different thicknesses are given in Fig. 3

and show a close correspondence with the experimental spectra i.e. (0.37 μmol/ml for a thickness of 100 nm, 3.7 μmol/ml for a thickness of 200 nm, 36 μmol/ml for a thickness of 300 nm and 334 μmol/ml for a thickness of 400 nm).

## Simulation

Numerical simulations were performed using commercial finite-difference time-domain (FDTD) software from Lumerical. Periodic boundary conditions were applied along the x- and y-axes and a perfectly matched layer (PML) was applied in the z-direction. The material data used for the Au pattern and the fused silica substrate were taken from Palik [54]. The dimensions used for the ASHs, and the periodicity, were based on those of the fabricated structure arrays. A plane wave source was set with its electric-field polarization parallel to the arms of the ASHs.

Using the FDTD software, the 17β-estradiol layer was modelled as two Lorentz oscillators in which the background relative permittivity of the analyte modifies the standard Lorentz resonance to produce a Fano-type resonance, as defined in Eq. (1) [10].

$$\varepsilon \left(f\right)=\varepsilon +\frac{\varepsilon_{L\omega_{L}^{2}}}{\omega_{L}^{2}-2i\delta_{L}\omega -\omega^{2}}$$ (1)

**Fig. 4:**
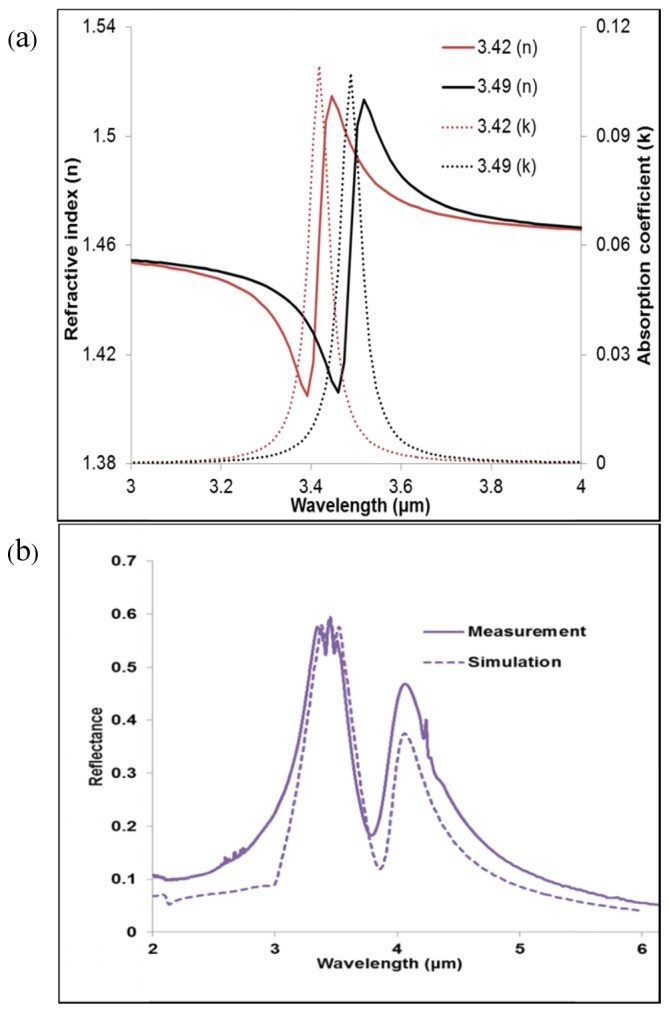
(a) Lorentz model for C-H bond resonances, (b) comparison of measurement for a solution concentration of 3.7 μmol/ml with simulation for a 200 nm thickness of E2. Note the close coincidence of the wavelengths of the molecular resonances with the shorter wavelength peak of the shifted ASH reflectance.

In this work, the background relative permittivity (Ɛ) of the analyte E2 is 2.40, obtained from the expression Ɛ = (n)^2^, where n (1.55) is used as the refractive index of the analyte. The applied refractive index is for the mid-infrared region and is close to the predicted index [55]. A Lorentz permittivity value (Ɛ_L_) of 0.0012 was used. A Lorentz linewidth value ($\delta_{L}$) of 2.2 x 10^12^ was used to provide resonance peaks that matched closely with the experimental measurements. The double resonance peaks produced by the C-H bonds have been modelled with Lorentz resonance angular frequencies (ω_L_) of 5.51 x 10^14^ radian/s and 5.4 x10^14^ radian/s which correspond to wavelengths of λ_1_ = 3.42 μm and λ_2_ = 3.49 μm respectively, as shown in Fig. 4(a)

.

The numerical models lead to Fano-type resonance, associated with the C-H bond vibration of the analyte, E2. This Fano resonance is formed by superimposition of the Lorentz resonance on a slowly varying dielectric background to produce the negative and positive changes observed in the refractive index of the E2 layer, while simultaneously producing a wavelength dependent absorption coefficient. The plots of the real part of the refractive index (*n*) and the absorption coefficient (*k*) for the analyte that result in Fano-resonance are as calculated from Eq. (1). A molecular resonance with a close match with the experimental data of 3.7 μmol/ml was achieved with our numerical simulations of a 200 nm thickness of E2, as shown in Fig. 4(b). The same design parameters were used to model E2 at different thicknesses 100 nm, 200 nm, 300 nm and 400 nm producing shifted reflectance peaks, as shown in Fig. 3. A three dimensional block with a square base area of 2 μm by 2 μm, covering a unit cell of the design at the given thicknesses, was applied for the numerical simulations of the analyte.

## Enhancement factor

The enhancement factor, *EF*, was calculated using the plots shown in Fig. 4. In this work, the expression of references [40] and [42] has been used to calculate the *EF* and it is defined as follows:

EF=ΔR2N2ΔR1N1. (2)

where N is the total number of molecules and ΔR is the relative change in the reflectance produced by the molecular resonance. N_1_ and ΔR_1_ correspond to measurements on unstructured surfaces, without ASH array patterning while N_2_ and ΔR_2_ correspond to the surface covered with an array of ASHs. It should be noted that it is possible to define the EF for the ASH structures by comparison with the change in reflectivity when the modelled analyte is deposited on either a uniformly gold-coated substrate or a fused silica substrate. Because of the high reflectivity of the gold surface, the amount of light that will be detected when the analyte is deposited on the gold will be much larger than for the case where the same amount of analyte is deposited on the fused silica substrate. It is our view that the uniform gold surface gives, therefore, a more appropriate basis of comparison.

**Fig. 5:**
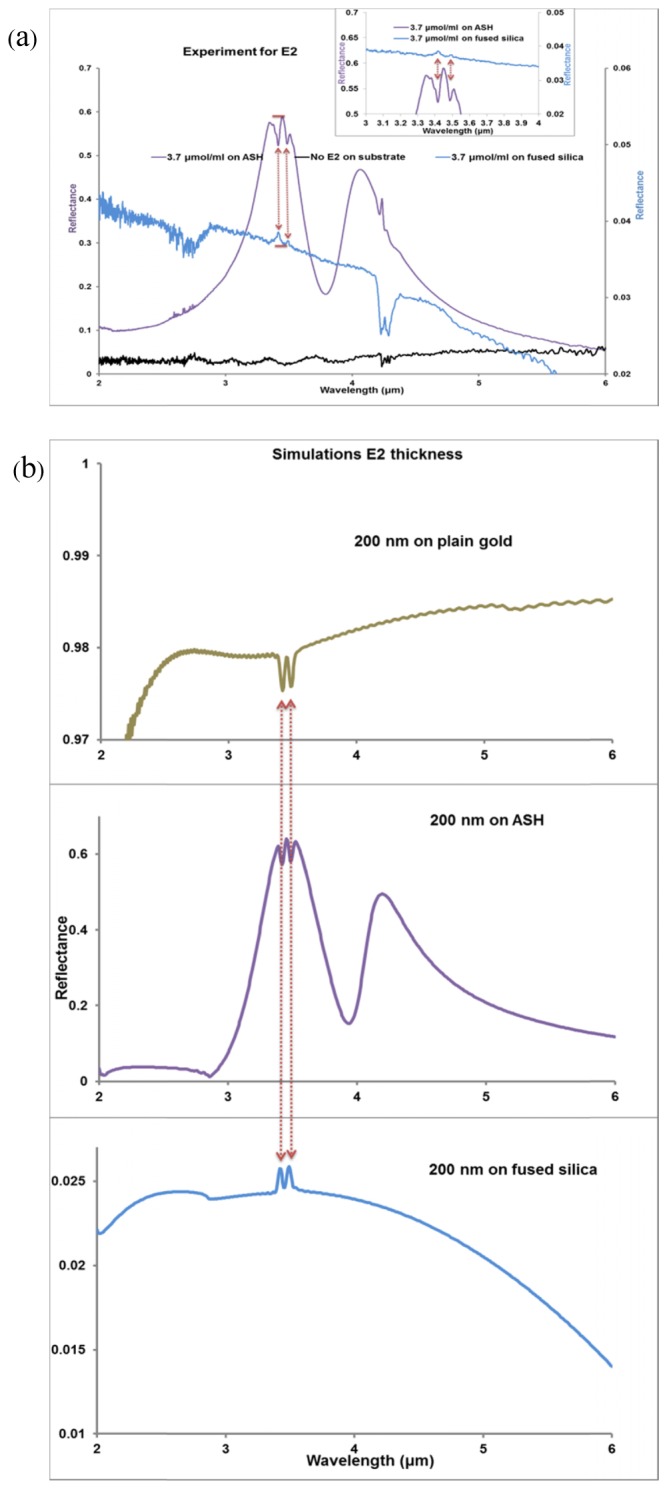
(a) FTIR reflectance spectra showing ripples from H_2_O vapour at between 2.5 μm and 3.0 μm, C-H molecular resonance bond stretch from 3.4 μm to 3.5 μm and CO_2_ at 4.2 μm for the 3.7μmole/ ml deposited on arrays of ASH and fused silica substrate, also is measurement of the fused silica substrate with no E2. The plots are displaced vertically for clarity (b) reflectance spectra from modelled 200 nm thickness of E2 on a plain fused silica substrate, plain gold and ASH nanoantenna.

The experiments were performed using solid E2 films deposited from four different E2 concentration of (0.37, 3.7, 36 and 334 μmol/ml). We have assumed that a homogenous thin film layer of the analyte, E2, covers the whole arrays of ASHs fabricated on a fused silica substrate, with each array covering a total area of 150 μm x 150 μm. From the four different experiments, enhancement of molecular resonances has been observed, in particular, for the concentration of 3.7 μmol/ml as shown in Fig. 2 and Fig. 5

. Figure 5(a) shows the reflectance spectrum obtained from fused a silica substrate with no deposited E2 on it, together with the reflectance spectrum for E2 deposited on the substrate from a solution with a concentration of 3.7 μmol/ml and the reflectance spectrum for E2 deposited from the same concentration on an array of ASHs. The deposition of the solid E2 film from the solution with this concentration left a film with a thickness of approximately 200 nm when measured with a surface profile measurement instrument. This thickness produced an absolute change in reflectance, ΔR_1,_ in the molecular resonance without ASHs of 0.00123 for the peak at 3.42 μm and; 0.00102 for the peak at 3.49 μm with 3.45 μm as the reference wavelength. Note that the molecular resonance peaks for E2 deposited on the silica substrate occur at exactly the same wavelengths as the inverted peaks (i.e. dips) in the reflectance spectrum for the E2 on ASH situation. The corresponding changes in absolute reflectance, ΔR_2_ with ASHs are: 0.06174 for the peak at 3.42 μm and 0.06138 for the peak at 3.49 μm, respectively. We estimate that the reflectance ratio: ΔR_2_/ ΔR_1_ has a value of 55 for both peaks. The values applied in the calculation have been obtained from the reflectance spectra of Fig. 5(a) for the molecular resonances. It should be noted that the enhancement factor is a maximum at the top of the peaks and different concentrations can move the overall plasmonic resonances off the molecular resonances.

The experimental results for a concentration of 3.7 μmol/ml were compared with simulation results for a thickness of 200 nm of the modelled analyte deposited on the three different surfaces over an area of 2 μm x 2 μm. Figure 5(b) shows the reflectance spectra obtained from the simulations. The values produced from the reflectance spectra of these simulations were used to calculate the *EF.*

**Fig. 6:**
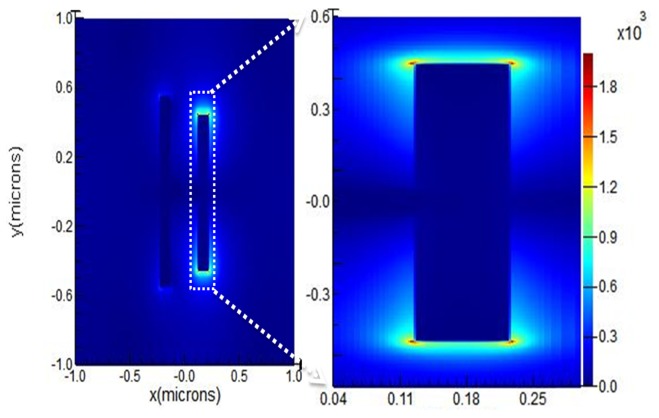
Absolute square magnitude of the E-field from simulation for the x-y axis in the presence of E2, with the dark red and dark blue colour showing the maximum and minimum value, respectively.

We consider the most effective resonance enhancement to occur for material that is in the area of the maximum E-field strength; such regions are commonly called ‘hot-spots’ [56–58]. The area of each of the four hot-spots is approximately 10 nm x 10 nm, as shown in Fig. 6

. The total hot-spot region is estimated by considering the approximate area where intensity falls to 1930/e^2^ i.e. 261 on the blue scale. This result has been obtained from the numerical simulation of the ASH nanoantenna reflectance in the analyte using |E|^2^/e^2^ as the criterion to define the extent of the hot spot. However, in the z-direction (above the metal surface of the ASH) the profiles shown in Fig. 6 initially rise and then fall to |E|^2^/e^2^ over the much greater distance of ~120 nm. We therefore estimate the volume of each of the hot spots to be 10 x 10 x 120 nm = 12000 nm^3^. Such hot-spots are typical features of sharp corners and edges and also nanoscale gaps in nanoantenna structures. For this design, the E-field is parallel to the arms of the ASH. The E-field magnitude plot was produced for the reflection resonance at a wavelength of 3.4 μm and it implies that there are four hot spots per unit cell, as shown in Fig. 6.

Given the array unit cell area of 2 x 2 μm and the analyte thickness of 200 nm, the volume of deposited analyte per ASH array unit cell is 8 x 10^−13^ cm^3^. The number of molecules N for the analyte E2, can then be calculated using equation: Number of molecules (N) = {(ρ x volume) x Avogadro’s Number} / molecular weight. The number of molecules, N_1_, in the volume of 8 x 10^−13^ cm^3^ is 2 x 10^9^ molecules when calculated without the presence of the ASH. The number of molecules, N_2_, per unit cell for the four hot-spots observed on each ASH nanoantenna is 1.2 x 10^5^ molecules. The ratio of the number of molecules is therefore: N_1_/N_2_ = 1.7 x 10^4^. A density (ρ) of 1.17 g/cm^3^ has been assumed for E2 [55]. Using Eq. (2), enhancement factors produced from ASH with reference to the two comparison materials, fused silica and plain gold, were calculated for absolute (A) and relative (R) changes in the reflectance at the molecular resonances, as shown in Table 1

**Table 1:** Enhancement factor based on the ratios of changes in reflectance of the molecular resonances and number of molecules

Parameters	Fused silica substrate (SiO_2_)	ASH	EF (SiO_2_:ASH)	Plain gold (Au)	ASH	EF (Au:ASH)
ΔR (Absolute)	0.001384	0.064506	7.9 x 10^5^	0.003403	0.063790	3.2 x 10^5^
ΔR (Relative)	0.053775	0.100544	3.2 x10^4^	0.003477	0.099425	4.9 x10^5^

.

The changes in reflectance, ΔV for a 200 nm thickness of the E2 analyte are as stated in Table 1 for the two molecular resonance peaks. The values for the calculation are taken from the reflectance spectra of Fig. 5(b) for the molecular resonances. We report a dip and peak at a wavelength between the two molecular resonances of the C-H bond. At a wavelength of 3.45 μm, changes in the refractive index of the surroundings due to the presence of the analyte cause a dip between the two molecular resonances at 3.42 μm and 3.49 μm for a fused silica substrate while a peak is observed for the same resonances for a plain gold surface and for the arrays of ASHs. The reflectance ratio of 53 estimated from simulation is in close comparison with the value of 55 obtained from experiment for the absolute changes in reflectance of the molecular resonances. Thus the enhancement factors which are inversely proportional to the estimated volume of influence by the nanoantennas, also depend on the substrate reference. In this work, the relative or absolute values of EF uses range from 3.2 x 10^4^ for the relative reflectance change referenced to silica to 7.9 x 10^5^ for the absolute reflectance change in referenced to gold.

## Conclusions

We have developed a novel metallic nanoantenna structure, the asymmetric H-shape (ASH) that has four sharp corners that enables sensitive assays of important hormones such as 17β-estradiol (E2). Plasmonic resonances induced in the nanoantenna can readily enhance the vibrational resonances of, for example, the C-H bond by a factor of as much as 10^5^, with a limit of detection (LOD) of 50 zeptomole per hot-spots of the ASH nanoantenna. A very sensitive biosensor has been achieved, with a sensitivity value of 2335 nm/RIU at a shifted wavelength of 4.87 μm and a figure of merit (FOM) of 8. The ASH nanoantenna has promise as a simple structure for the sensitive detection of various hormones. The important demands for detection of E2 in biomedical applications and water treatment analysis highlight the importance of using the simplest and straightforward means of performing assays. We have modeled this molecular resonance using a Lorentzian model that gives a close match with the spectral resonances observed experimentally. The narrow gap in the split ASH structure is primarily relevant for a situation where a significant part of the incident light is polarized orthogonally to the dipole arms and therefore has an electric field vector that is directed across the gap. The split ASH structure, with suitably chosen dimensions, therefore has the potential to give substantially polarization independent operation. This possibility will be the subject of a subsequent paper.
